# A Flexible Wearable Glucose Sensor for Noninvasive Diabetes Screening: Functional Equivalence and Model Interpretability

**DOI:** 10.3390/bios16040214

**Published:** 2026-04-10

**Authors:** Wenhan Xie, Jinqi Wang, Hao Liu, Shuo Chen, Peng Wang, Yumei Han, Xianxiang Chen, Zhen Fang, Zhan Zhao, Guohong Zhang, Xiuhua Guo

**Affiliations:** 1 Beijing Key Laboratory of Environment and Aging, Department of Epidemiology and Health Statistics, School of Public Health, Capital Medical University, Beijing 100069, China; xiewenhan@mail.ccmu.edu.cn (W.X.); wangjinqi@ccmu.edu.cn (J.W.); 6hhhhhh@mail.ccmu.edu.cn (H.L.); 2 Beijing Physical Examination Center, Beijing 100050, China; cs@bjtjzx.com (S.C.); hanyumei@bjtjzx.com (Y.H.); zgh@bjtjzx.com (G.Z.); 3 Aerospace Information Research Institute, Chinese Academy of Sciences, Beijing 100094, China; wangpeng01@aircas.ac.cn (P.W.); chenxx@aircas.ac.cn (X.C.); zfang@mail.ie.ac.cn (Z.F.); zhaozhan@mail.ie.ac.cn (Z.Z.); 4 National Institute for Data Science in Health and Medicine, Capital Medical University, Beijing 100069, China; 5 School of Medical and Health Sciences, Edith Cowan University, Perth 6027, Australia

**Keywords:** noninvasive glucose monitoring, wearable device, type 2 diabetes mellitus, machine learning, population screening

## Abstract

Real-world evidence for wearable noninvasive glucose monitoring (NIGM) remains limited. To evaluate the functional equivalence of a wearable NIGM device and explore its utility for T2DM and prediabetes screening. In this multicenter study, 12-h daytime glucose profiles obtained by a flexible reverse iontophoresis-based electrochemical sensor were compared with capillary glucose using functional equivalence. Subgroup analyses were conducted. Screening models of T2DM and prediabetes were developed using elastic net and Logistic regression. A total of 135 participants (mean age 35.3 years; 60.0% female) were included, and no serious device-related adverse events were reported. Compared to the capillary measurements, functional equivalence was confirmed (*T* = −6.537 < threshold = −2.081) in the general population but not in older adults or T2DM patients. The T2DM noninvasive screening model demonstrated discrimination and reclassification performance comparable to those of the capillary-based model (AUC: 0.906 vs. 0.850, NRI: 0.044, IDI: −0.078, *p* > 0.05). Functional principal component scores facilitated the identification of prediabetes (AUC = 0.760). The device demonstrated acceptable accuracy and functional equivalence with reference methods. Its capability to detect T2DM and early glycemic anomalies supports its feasibility as a wearable, interpretative adjunct tool for large-scale screening in free-living populations.

## 1. Introduction

Diabetes imposes a substantial and growing global public health burden. In 2024, an estimated 589 million adults aged 20 to 79 years were living with diabetes, and this number is projected to reach 853 million by 2050 [1]. Notably, a substantial proportion remains undiagnosed, and delayed detection of diabetes is associated with a greater risk of microvascular and macrovascular complications, which underscores the need to expand population-level screening and longitudinal monitoring [2,3]. International consensus statements, including those from the American Diabetes Association, recommend that continuous glucose monitoring (CGM) metrics be considered to characterize daily glycemia and inform treatment decisions [4]. However, adherence to venous sampling or fingerstick capillary testing is limited by pain and inconvenience, and CGM uptake in routine care is constrained by cost, coverage, and supply, as well as wearability, which restricts scalable access to high-quality continuous glucose data, particularly in asymptomatic or low-risk populations [5,6,7].

Against this backdrop, noninvasive glucose monitoring (NIGM) has attracted attention because it avoids skin puncture and supports long-term wear [8,9]. Most optical, physiologic-signal-based, thermal, and electromagnetic approaches estimate glucose indirectly from surrogate signals. These techniques generally require calibration and can vary with individual physiology and environmental conditions [10,11]. An alternative strategy is to extract interstitial fluid (ISF) across intact skin and measure glucose electrochemically [12,13]. ISF glucose closely follows blood glucose with only a short, physiology-dependent lag of a few minutes, suggesting that if extraction efficiency and calibration are well controlled, transdermal ISF sampling can generate numerically interpretable glucose readouts [14,15]. In addition, many noninvasive glucose monitoring systems are designed mainly for hospital or other supervised settings, which limits their usefulness for population-level screening and glycemic monitoring in free-living conditions, where comfort, economy, and portability are as important as measurement accuracy for users [16].

The current evaluation framework for NIGM technologies primarily relies on point-to-point accuracy metrics, such as the mean absolute relative difference (MARD) [17]. Due to physiological lag in glucose measurement and the susceptibility of noninvasive interfaces to microenvironmental variations, isolated point-in-time comparisons may lead to overestimation of errors, thereby obscuring the sensor’s capability to capture dynamic glucose fluctuations [18]. More significantly, for large-scale population screening and early diabetes risk identification, clinical value may rely more heavily on assessing glucose fluctuation morphology and trend consistency [19]. Consequently, novel evaluation dimensions—such as functional equivalence testing—are required to assess the screening efficacy of noninvasive devices in real-world settings at the overall curve level [20].

Our research team has previously developed a NIGM device incorporating flexible electrochemical sensing and reverse iontophoresis (RI), with preliminary on-body studies confirming its accuracy [21,22]. However, its performance may be constrained when applied in uncontrolled, free-living environments. Consequently, our study aims to evaluate the device’s performance in real-world short-term glucose monitoring and disease screening, with specific objectives to: (1) explore functional equivalence between noninvasive and capillary glucose measurements and (2) analyze the device’s performance in screening participants at high risk for type 2 diabetes mellitus (T2DM) and prediabetes.

## 2. Materials and Methods

### 2.1. Study Setting and Population

This multicenter study recruited 150 adults from the University of Chinese Academy of Sciences and the Beijing Physical Examination Center in China, between 13 January and 15 June 2025. Eligible participants included adults with normal or abnormal glycemic status, without major surgery or trauma within the preceding three months, and without known secondary causes of glucose abnormalities. After excluding one participant with unavailable fasting venous glucose due to hypoglycemia and 14 participants with incomplete or invalid data due to operational problems, 135 participants were included for the main analyses.

The study was approved by the Ethics Committee of Capital Medical University (2024SY034) and the Beijing Physical Examination Center (BJTJZX-005), and was registered with the China Clinical Trial Registry (ChiCTR2400093042). All participants provided written informed consent.

### 2.2. Wearable NIGM Devices and Glucose Measurements

The factory-calibrated wearable NIGM device, MicroTED, developed jointly by the University of Chinese Academy of Sciences and Capital Medical University, was a flexible wearable system that integrated RI for ISF extraction with a low-cost cellulose paper-based electrochemical glucose sensor [21]. The system comprised a disposable sensing patch and a reusable electronic module featuring Bluetooth communication and a 3.7 V/300 mAh lithium-ion battery, enabling over 12 h of continuous operation. Based on prior design of experiments optimization, the RI extraction was configured with a pulsed direct current waveform delivering a 100 µA microcurrent. The specific parameters were set at a 12 V maximum amplitude, 1 kHz frequency, and 10% duty cycle to ensure efficient ISF sampling while minimizing skin irritation. To enhance measurement stability against skin matrix effects, the device employed a real-time monitoring module that continuously measured the surface water content of the extraction electrode, extraction current, and extraction voltage in situ. A Kalman filter algorithm was utilized in the post-signal processing of these sensor data to effectively remove noise and ensure the reliability of the extraction status. Previous pilot validation in 6 volunteers (4.5-h duration) demonstrated high agreement with a commercial glucometer. Further mechanisms could be found in the previous report [21].

Continuous noninvasive monitoring was initiated after an overnight fast (>8 h) and spanned a 12-h daytime period with readings obtained approximately every 30 min. To validate glycemic trends, concurrent reference capillary glucose levels were measured 6 times daily (pre- and 2-h post-prandial) using commercial glucometers (ACCU-CHEK Performa, Basel, Switzerland). Throughout the session, participants maintained unrestricted daily activities to simulate a free-living environment.

### 2.3. Definition of T2DM and Prediabetes Status

T2DM risk status was defined based on self-reported diabetes status and current use of antidiabetic medication, in combination with venous fasting plasma glucose ≥ 7.0 mmol/L or glycated hemoglobin (HbA1c) ≥ 6.5%, which was used for risk screening [23]. To evaluate the capability of MicroTED in early risk identification, prediabetes was defined as venous fasting plasma glucose between 6.1 and 6.9 mmol/L or HbA1c between 5.7% and 6.4%, without T2DM [24].

### 2.4. Covariates and User Experience

Covariates included sociodemographic factors (age, sex, marital status, education, and ethnicity), lifestyle behaviors (smoking, alcohol consumption, sleep duration, napping, staying up late, regular exercise, and sweet preference), anthropometric measures (body mass index [BMI], waist-to-hip ratio [WHR], systolic blood pressure, diastolic blood pressure, and resting pulse), disease history (self-reported hypertension, and family history of T2DM), and laboratory factors (venous fasting glucose and HbA1c).

Participants were also asked to report adverse events or any device-related discomfort during the study period. User experience was assessed using a five-point comfort rating scale, which was summarized as overall comfort and discomfort. All measurements underwent strict quality-control procedures to ensure data reliability. Detailed variable definitions, comfort rating procedures, and quality control methods are provided in the Supplementary Materials.

### 2.5. Statistical Analysis

Continuous variables were reported as mean ± standard deviation (SD) and compared using Student’s *t*-test. Categorical variables were presented as counts and percentages and compared using the *χ*^2^ test. The accuracy of noninvasive glucose vs. venous glucose measurements was evaluated using the mean absolute relative differences (MARD) and Clarke error grid analyses.

#### 2.5.1. Functional Equivalence Testing for Short-Term Glycemic Profile

Each participant contributed multiple noninvasive glucose measurements (median 22; range 7–27) and up to 6 paired capillary glucose values during the monitoring period (Figure S1). To account for irregular sampling intervals, individual glucose trajectories were reconstructed using functional principal component analysis via the conditional expectation method [25]. Eigenfunctions explaining >99% of the total variance were retained to describe the major temporal patterns in glucose variation [26].

Equivalence between noninvasive and capillary glucose curves was assessed using a functional equivalence test based on maximal deviation [20]. Equivalence bounds were defined as ±1.11 mmol/L when the capillary glucose was <5.6 mmol/L and ±20% otherwise [27,28]. A test statistic was constructed as the maximum deviation of glucose difference beyond the prespecified bounds, and functional equivalence was declared when the observed statistic fell below the 5th percentile of the bootstrap distribution. More details are provided in the Supplementary Materials.

To evaluate the robustness, subgroup analyses were performed stratified by age (<45 years vs. ≥45 years), sex (male vs. female), T2DM status (yes vs. no), and trial site (Subcenter A vs. Subcenter B). Sensitivity analyses were conducted by re-evaluating equivalence using alternative, tighter (±15%/0.83 mmol/L) and wider (±30%/1.67 mmol/L) acceptance boundaries.

#### 2.5.2. Noninvasive Model Development for T2DM and Prediabetes Risk Screening

Participants were randomly assigned to a training set and a validation set at a 7:3 ratio using stratified sampling to ensure a balanced distribution of T2DM cases. Variable selection was performed using elastic net regularization, combining *L*_1_ penalty and *L*_2_ penalty to select features with the optimal mixing percentage (*α*) and penalty strength (*λ*) determined via 10-fold cross-validation. Multivariable Logistic models were developed to identify individuals at higher risk of T2DM to explore the potential screening utility of noninvasive glucose measurements.

Model discrimination was assessed using the area under the receiver operating characteristic curve (AUC) with 95% confidence intervals (*CI*s). A nomogram was developed based on the final model to facilitate individualized assessment of T2DM risk and decision curve analysis (DCA) to explore utility. To compare screening performance, a parallel capillary-based model was developed, and the incremental value of noninvasive glucose was assessed using net reclassification improvement (NRI) and integrated discrimination improvement (IDI). The Shapley Additive exPlanations (SHAP) analysis was employed to visualize feature contributions.

Given the limited sample size of T2DM cases, we used 10-times repeated 5-fold cross-validation to revalidate the robustness of screening models. To further validate the MicroTED utility for detecting occult T2DM, which was a critical real-world screening scenario, we performed a targeted analysis excluding participants with a self-reported history of diabetes or current use of hypoglycemic agents. In the “undiagnosed population” (*n* = 125), the discrimination performance of the noninvasive model was re-evaluated to confirm its robustness. Furthermore, to assess the potential for early identification, a secondary screening model was constructed to distinguish individuals with prediabetes among those excluding T2DM cases.

Missing values in questionnaire variables were imputed using multiple imputation by chained equations. All statistical analyses were performed using R software (version 4.4.1), with significance set at *p* < 0.05.

## 3. Results

### 3.1. Participant Characteristics

Among the 135 participants, 21 (15.6%) individuals were diagnosed with T2DM. The mean age was 35.34 (*SD:* 14.78) years, and 81 (60.0%) participants were female. As shown in Table 1, participants with T2DM were older, married, had lower educational attainment, reported never staying up late, and were more likely to have hypertension compared with glucose-normal individuals (all *p* < 0.05). No serious device-related adverse events were observed. 110 (81.5%) participants reported the device as overall comfortable; only 8 (5.9%) individuals reported discomfort. Based on the 135 paired observations, the overall MARD was 15.13% (95%*CI*: 12.38%, 18.39%), specifically 14.61% (95%*CI*: 11.83%, 17.97%) for fasting venous glucose < 7.0 mmol/L and 26.40% (95%*CI*: 15.85%, 35.34%) for ≥7.0 mmol/L. All fasting glucose pairs were within Clarke’s clinically acceptable zones (Zone A: 114/135, 84.44% [95%*CI*: 77.39%, 89.59%]; Zone B: 21/135, 15.56% [95%*CI*: 10.41%, 22.61%]), as shown in Figure S2.

### 3.2. Equivalence of Short-Term Glycemic Profiles

**Figure 1:**
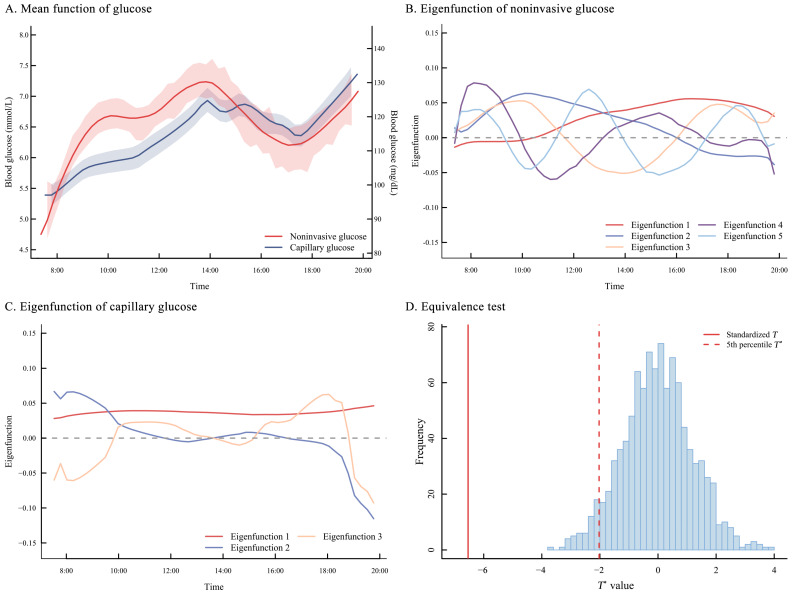
Functional representations and equivalence test of glucose profiles. Notes: (**A**) was the mean functions of noninvasive and capillary glucose, with shaded areas indicating corresponding 95%*CI*s; (**B**) was the first five eigenfunctions of noninvasive glucose; (**C**) was the first three eigenfunctions of capillary glucose; and (**D**) was standardized observed statistic (*T*) and the bootstrap distribution of the test statistic (T*) in functional equivalence testing.

The mean functions of noninvasive and capillary glucose demonstrated broadly similar temporal trends, characterized by an initial rise, a subsequent decline, and a later increase (Figure 1A). The reconstructed noninvasive and capillary glucose curves were adequately represented by five eigenfunctions (explaining 99.86% of the total variance) and three eigenfunctions (explaining 99.27% of the variance), respectively (Figure 1B,C). The first eigenfunctions of both noninvasive and capillary glucose curves displayed relatively stable trajectories with slight upward trends over the 12 h; the second eigenfunction of the noninvasive glucose transitioned from positive to negative around 16:00, indicating the shift from morning to evening; the third eigenfunction exhibited alternating positive and negative phases, reflecting localized fluctuations; the others captured the remaining short-term variations. Corresponding eigenvalues and cumulative variance proportions are listed in Table S1.

The functional equivalence test rejected the null hypothesis (observed *T* = −6.537 < threshold *T* = −2.081), indicating that noninvasive and capillary glucose curves were statistically equivalent (Figure 1D).

Subgroup analyses revealed that functional equivalence was supported among participants aged < 45 years, glucose-normal individuals, both male and female, and across all trial sites. In contrast, equivalence could not be established in participants aged ≥ 45 years (observed *T* = −0.957 > threshold *T* = −1.174) or those with T2DM (observed *T* = 2.760 > threshold *T* = −2.672), as seen in Figure S3. Results remained similar when using alternative bounds: for ±15% (0.83 mmol/L), the observed *T* was −2.651 (threshold *T* = −2.081); and for ±30% (1.67 mmol/L), the observed *T* was −13.733 (threshold *T* = −1.156), indicating that equivalence was supported under all alternative specifications.

### 3.3. Noninvasive Model of Disease Screening

#### 3.3.1. Noninvasive Screening for T2DM Risk

**Figure 2:**
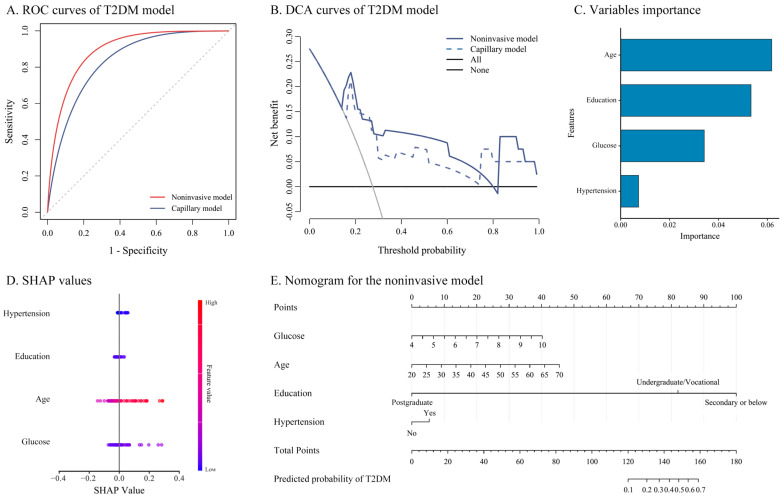
Screening models for T2DM risk. Notes: (**A**) was the ROCs of noninvasive and capillary models; (**B**) was the DCA curves of the noninvasive and capillary model; (**C**) was SHAP value ranking of the variables in the noninvasive model; (**D**) was the SHAP honeycomb diagram of the noninvasive model; and (**E**) was the nomogram of the noninvasive model, including the fasting glucose, age, marital status, and education. Abbreviations: ROC, receiver operating characteristic; T2DM, type 2 diabetes mellitus; DCA, decision curve analysis; SHAP, SHapley Additive exPlanations.

Baseline characteristics of the training and validation sets are shown in Table S2. Under the mixing percentage (*α* = 0.6) and penalty parameter (*λ* = 0.149) corresponding to the one-standard-error criterion, we selected four variables: noninvasive fasting glucose, age, education, and hypertension (Figure S4). As seen in Figure 2A and Table 2, the AUCs of the noninvasive model (0.906 [95%*CI*: 0.800, 1.000]) and the capillary model (0.850 [95%*CI*: 0.706, 0.993]) did not differ significantly (*p* = 0.124). Consistently, no statistically significant differences were observed for NRI (0.044, *p* = 0.566) or IDI (−0.078, *p* = 0.073). Additional information on the validation and training sets is provided in Table S3.

The accuracy, sensitivity, and specificity were 0.825 (95%*CI*: 0.672, 0.927), 0.889 (95%*CI*: 0.518, 0.997), and 0.806 (95%*CI*: 0.625, 0.925), respectively. DCA indicated that the noninvasive model yielded a higher net benefit than either treating all or treating none (Figure 2B). According to the feature importance ranking (Figure 2C), noninvasive fasting glucose was identified as a key predictor, ranking third after age and education. Elevated glucose levels contributed to the increased risk of T2DM (Figure 2D). The nomogram of the noninvasive model is shown in Figure 2E.

The results of repeated cross-validation analysis were similar to the main analyses. Specifically, the AUCs of the noninvasive and capillary models were 0.844 (95%*CI*: 0.814, 0.873) and 0.830 (95%*CI*: 0.797, 0.863), respectively (Table S4).

#### 3.3.2. Screening Performance in the Undiagnosed Population

As for participants without self-reported diabetes or hypoglycemia medication (*n* = 125), the AUC of the noninvasive T2DM model was 0.794 (95%*CI*: 0.677, 0.912), which remained similar to the capillary model (AUC = 0.772, *p* = 0.619; NRI = 0.044, *p* = 0.295; IDI = −0.027, *p* = 0.439; Table S5).

#### 3.3.3. Noninvasive Screening for Prediabetes Risk

Excluding individuals with diabetes, we constructed a screening model for prediabetes (*n* = 114), randomly dividing the dataset into training and validation sets at a 7:3 ratio (Table S6). Under the mixing percentage (*α* = 0.5) and penalty parameter (*λ* = 0.171) corresponding to the one-standard-error criterion, we selected six variables: second and third principal components of noninvasive glucose, age, hypertension, ethnicity, and alcohol consumption (Figure S5). The AUC, accuracy, sensitivity, and specificity were 0.760 (95%*CI*: 0.572, 0.948), 0.735 (95%*CI*: 0.556, 0.871), 0.889 (95%*CI*: 0.518, 0.997), and 0.680 (95%*CI*: 0.465, 0.851) (Table S7).

## 4. Discussion

The multicenter study evaluated the performance and screening utility of a novel wearable NIGM system, MicroTED, under free-living conditions. Our findings demonstrate that the device offered acceptable measurement accuracy and high comfort. We discovered that the short-term noninvasive glycemic profiles were statistically equivalent to reference capillary glucose trends in the general population, although discrepancies were observed in older adults and patients with T2DM. Furthermore, the MicroTED-based screening model achieved excellent discrimination for identifying T2DM risk, comparable to the capillary model, and demonstrated potential for early prediabetes detection using dynamic glucose trajectory features. Taken together, these results indicate that MicroTED serves as a promising, accessible tool for large-scale T2DM screening and early risk identification.

T2DM and prediabetes are often detected only after years of unrecognized dysglycemia, and global estimates suggest that roughly 43% of adults living with diabetes remain undiagnosed [2,29]. Prolonged hyperglycemia can precede clinical diagnosis by about 4–7 years, during which time people may already develop microvascular damage and present with retinopathy, neuropathy, or nephropathy at their first clinical contact [30]. These observations point to a substantial window in which earlier detection and intervention could prevent or delay complications. In principle, regular glucose testing could help identify high-risk individuals earlier. Still, adherence to repeated capillary glucose testing is often poor because testing is painful, inconvenient, and disruptive to daily life [31]. In routine care, glucose monitoring therefore depends on a combination of fingerstick testing and CGM [4]. CGM uses a subcutaneous sensor to measure interstitial glucose continuously, provides detailed glucose profiles, and is recommended in contemporary standards of care for many people with diabetes, yet use in everyday practice remains limited because devices are costly, require prescription and user training, and rely on transcutaneous sensors that can cause discomfort, skin reactions, and alarm burden [32,33]. To address both access and wearability, we developed MicroTED. This soft, patch-like, entirely noninvasive glucose monitoring device uses reverse iontophoresis with on-skin electrochemistry rather than a subcutaneous sensor. It is designed for comfortable daytime wear without skin puncture [21]. In our study, the device was generally well tolerated, and most participants reported that it was comfortable to wear and did not interfere with usual daily activities. Additionally, our findings show that the wearable device based on reverse iontophoresis with on-skin electrochemistry (RIOSE) can provide both fasting glucose values and 12-h daytime glycemic profiles without skin penetration, with performance comparable to invasive approaches. In addition, the device performed well in screening for T2DM. These findings suggest a potential role for entirely noninvasive glucose monitoring in population screening for previously unrecognized diabetes or high-risk dysglycemia, and in longitudinal risk monitoring and day-to-day feedback, particularly for people with limited access to conventional invasive testing.

Noninvasive glucose monitoring has been explored for several decades using electrochemical, optical, and electromagnetic approaches, but many devices tested to date have shown only moderate accuracy and practical limitations when evaluated in ambulatory humans [34,35]. RI devices, such as the GlucoWatch biographer, were among the earliest systems to provide automated on-body readouts by extracting interstitial glucose through intact skin. Yet, they required frequent fingerstick calibration and showed median relative absolute differences of about 16–21% versus reference glucose, with poorer performance during hypoglycemia [36,37]. Regulatory and clinical reports also described an overall lag of roughly 15–20 min between GlucoWatch readings and blood glucose and iontophoresis-related skin irritation, which together limited user acceptance and contributed to withdrawal from the market [36,37,38]. More recent optical and electromagnetic noninvasive devices, including Raman spectroscopy-based systems, have reported mean absolute relative differences of about 10–14%, with most paired values in Clarke or consensus error grid zones A and B [10,16,39]. However, these devices have mainly been evaluated as research prototypes in small clinical or laboratory studies or protocolized home-use trials (typically ≈20–160 participants, often with established diabetes), usually requiring hours-long individualized calibration and being assessed over limited monitoring periods ranging from a few days to several weeks, rather than in large, unsupervised everyday use [10,39]. In this context, given that the MicroTED noninvasive device provided glucose readings comparable to capillary measurements, it may offer a practical option for day-to-day self-monitoring and for noninvasive identification of individuals at increased risk for T2DM. However, it is essential to note that laboratory-based testing will still be required to confirm T2DM and to guide treatment decisions.

In subgroup analyses, device performance was broadly similar across sex and study centers, with fasting MicroTED readings and 12-h daytime profiles showing comparable agreement with invasive reference measures. These findings support the robustness of the RIOSE approach across different populations and implementation sites. By contrast, both absolute accuracy and curve-level concordance were attenuated among participants with established T2DM and in older adults. This pattern is biologically plausible. Chronic hyperglycemia in T2DM is associated with structural and functional changes in the skin, including reduced stratum corneum hydration, thickening of the stratum corneum, altered barrier function, and impaired cutaneous microcirculation, all of which can modify interstitial fluid composition and hinder transdermal extraction [40,41]. Aging itself is also accompanied by a decline in skin barrier function, decreased hydration and elasticity, and microvascular dysfunction, which may further reduce the efficiency and stability of RI-based sampling [41,42]. In addition, the physiological lag time between interstitial fluid and blood glucose levels is increased when the rate of change in blood glucose exceeds the limited rate of capillary diffusion [43]. People with long-standing T2DM often have greater glycemic variability and may receive glucose-lowering therapies that induce rapid glucose excursions, as well as a reduced rate of glucose transport into the interstitial fluid of subcutaneous tissue compared to healthy individuals [44,45]. All of these factors may exacerbate discrepancies between a transdermal signal and venous or capillary reference values. Notably, when we restricted analyses to participants without known T2DM at baseline, agreement between noninvasive and invasive measures improved compared with the overall cohort. Taken together, these findings suggest that this entirely noninvasive patch may be most informative for describing daytime glucose patterns and for identifying adults without known T2DM who may have previously unrecognized T2DM or high-risk dysglycemia and therefore warrant confirmatory testing. Notably, because only a small number of older adults and participants with T2DM were included, statistical power for these subgroups was limited. Device performance in these groups remains uncertain and should be evaluated in larger, dedicated studies.

The robust discrimination capability of the noninvasive model for T2DM, which proved statistically comparable to capillary models even in undiagnosed populations, highlights its potential as a barrier-free screening tool. The integration of SHAP analyses further confirmed that the model captures genuine physiological risk patterns driven by glucose levels, age, and education, which is similar to previous studies [46,47]. Uniquely, the continuous nature of NIGM allowed for the extraction of dynamic trajectory features, namely functional principal components, facilitating the identification of prediabetes, which often presents with subtle glycemic variability preceding fasting hyperglycemia [48,49]. Specifically, the “positive to negative” shift in the second functional principal component of noninvasive glucose indicates differences in glucose changes from morning to evening, which might reflect short-term rhythmic variations; the “positive-negative-positive” oscillations in the third principal component might reflect meal-driven excursions and postprandial recovery, while its derivative function reflects the rates of fluctuation and recovery. Notably, although the sensitivity of the prediabetes model was high, specificity and PPV were modest, which might limit the screening efficiency of the model. From a clinical perspective, an entirely noninvasive patch such as MicroTED is best considered an adjunct to standard laboratory and capillary-based glucose testing rather than a replacement. In adults without known T2DM, particularly those who feel well but have risk factors such as a family history of diabetes or excess adiposity, brief daytime wear in primary care or community settings could provide a simple snapshot of glucose behavior and help identify individuals who should be referred for diagnostic evaluation or closer surveillance [23,50]. Noninvasive patches could also be incorporated into digital health or population risk-assessment programs to extend glucose profiling beyond people who currently receive CGM or perform frequent self-monitoring, especially where access to invasive technologies is limited. These potential applications should be evaluated in pragmatic studies that embed the patch into routine care and examine its impact on referral patterns, time to diagnosis, patient experience, and costs compared with existing strategies.

This study has several strengths. First, its multicenter design and the use of venous plasma glucose as the laboratory reference enhance the reliability and generalizability of the findings. Second, we comprehensively evaluated device performance by combining fasting glucose measurements with a 12-h continuous monitoring period and employed advanced functional data analysis to address irregular and sparse sampling, enabling a more accurate comparison of glucose trajectories.

Nevertheless, several limitations should be acknowledged. First, the study population was relatively young, and the number of participants with T2DM was small, which may have reduced generalizability and partly accounted for the lower accuracy and reduced equivalence observed in older adults (≥45 years) and individuals with T2DM. Future studies involving more heterogeneous and clinically representative populations are warranted. Second, the monitoring duration was relatively short. Due to the general-population nature of our research and constraints in device availability, only a single 12-h daytime session was recorded, without nocturnal measurements or repeated assessments across multiple days. Longer-term monitoring will be essential to characterize glycemic patterns better. Third, because prolonged venous blood sampling is challenging in free-living individuals, capillary glucose measurements served as the reference. Although this method is widely adopted and clinically validated for self-monitoring, the substitution may nonetheless introduce measurement variability. In addition, inter-individual differences in skin physiology, including factors such as temperature, sweating, and hydration status that may influence RI-based glucose extraction [51], were not assessed and require further study. Finally, given the sparse nature of our current reference glucose data, we plan to systematically collect high-frequency blood glucose measurements in future studies, which will allow us to accurately quantify the specific, dynamic physiological lag times between interstitial fluid and blood glucose across varying glycemic states, and to explore the integration of predictive algorithms to dynamically align noninvasive signals for real-time glucose monitoring.

## 5. Conclusions

This multicenter study validates the performance of the MicroTED system, an entirely noninvasive glucose monitor integrating reverse iontophoresis and electrochemical sensing. The device demonstrated acceptable accuracy and functional equivalence to capillary glucose trends in the general population. Notably, the noninvasive screening model achieved robust discrimination for T2DM risk and showed promise for early prediabetes identification through dynamic glycemic features. These findings suggest the device serves as a painless and accessible adjunct tool for population-level screening and risk identification in free-living settings, not a replacement modality for standard glucose monitoring or confirmatory laboratory testing. Future research should focus on optimizing algorithms for older adults and evaluating the system’s long-term health economic impact in diverse populations.

## Figures and Tables

**Table 1:** Baseline characteristics for individuals.

Characteristics	Overall (*n* = 135)	T2DM		*p*-Value
		No (*n* = 114)	Yes (*n* = 21)	
Trial site				0.069
Subcenter A	82 (60.74)	65 (57.02)	17 (80.95)	
Subcenter B	53 (39.26)	49 (42.98)	4 (19.05)	
Age, years	35.34 (14.78)	32.64 (12.77)	50.00 (16.57)	<0.001
Sex				0.594
Male	54 (40.00)	44 (38.60)	10 (47.62)	
Female	81 (60.00)	70 (61.40)	11 (52.38)	
Marital status				0.003
Married	54 (40.00)	39 (34.21)	15 (71.43)	
Other	81 (60.00)	75 (65.79)	6 (28.57)	
Education				<0.001
Secondary or below	20 (14.81)	9 (7.89)	11 (52.38)	
Undergraduate or vocational	67 (49.63)	58 (50.88)	9 (42.86)	
Postgraduate	48 (35.56)	47 (41.23)	1 (4.76)	
Ethnicity				0.493
Han	124 (91.85)	106 (92.98)	18 (85.71)	
Other ethnic minorities	11 (8.15)	8 (7.02)	3 (14.29)	
Smoking				>0.999
Yes	12 (8.89)	10 (8.77)	2 (9.52)	
No	123 (91.11)	104 (91.23)	19 (90.48)	
Alcohol consumption				1.000
Yes	6 (4.44)	5 (4.39)	1 (4.76)	
No	129 (95.56)	109 (95.61)	20 (95.24)	
Sleep duration				0.979
7–8 h per day	80 (59.26)	67 (58.77)	13 (61.90)	
Others	55 (40.74)	47 (41.23)	8 (38.10)	
Napping				0.530
≤2 days per week	85 (62.96)	70 (61.40)	15 (71.43)	
≥3 days per week	50 (37.04)	44 (38.60)	6 (28.57)	
Staying up status				0.007
Never	34 (25.19)	23 (20.18)	11 (52.38)	
Sometimes	35 (25.92)	32 (28.07)	3 (14.29)	
Always	66 (48.89)	59 (51.75)	7 (33.33)	
Exercise				0.673
Never	27 (20.00)	24 (21.05)	3 (14.29)	
Sometimes	60 (44.44)	49 (42.99)	11 (52.38)	
Always	48 (35.56)	41 (35.96)	7 (33.33)	
Sweety preference				0.230
Yes	37 (27.41)	34 (29.82)	3 (14.29)	
No	98 (72.59)	80 (70.18)	18 (85.71)	
Venous glucose, mmol/L	5.11 (0.94)	4.85 (0.45)	6.48 (1.56)	<0.001
Noninvasive glucose, mmol/L	5.11 (1.22)	4.96 (1.19)	5.95 (1.04)	<0.001
HbA_1c_, %	5.81 (0.90)	5.53 (0.43)	7.33 (1.20)	<0.001
BMI, kg/m^2^	23.28 (5.45)	22.91 (5.39)	25.27 (5.49)	0.068
WHR, cm/cm	0.83 (0.09)	0.82 (0.09)	0.86 (0.11)	0.093
Pulse, bpm	73.36 (9.37)	73.65 (9.68)	71.81 (7.51)	0.410
Hypertension				0.002
Yes	15 (11.11)	8 (7.02)	7 (33.33)	
No	120 (88.89)	106 (92.98)	14 (66.67)	
Family history of T2DM				0.173
Yes	12 (8.89)	8 (7.02)	4 (19.05)	
No	123 (91.11)	106 (92.98)	17 (80.95)	
Perceived comfort of the device				0.709
Overall comfort	110 (81.48)	94 (82.46)	16 (76.19)	
Discomfort	8 (5.93)	7 (6.14)	1 (4.76)	
Missing	17 (12.59)	13 (11.40)	4 (19.05)	

Notes: Data are presented as mean ± SD or *n* (%). Abbreviations: T2DM, type 2 diabetes mellitus; BMI, body mass index; WHR, waist-to-hip ratio.

**Table 2:** Comparison of noninvasive and capillary models for T2DM.

	Estimate	95%*CI*	*p*-Value
AUC			0.124
Capillary model	0.850	0.706–0.993	
Noninvasive model	0.906	0.800–1.000	
NRI			0.566
Capillary model	Ref.		
Noninvasive model	0.044	−0.106–0.194	
IDI			0.073
Capillary model	Ref.		
Noninvasive model	−0.078	−0.163–0.007	

Notes: the capillary and noninvasive models included fasting glucose, age, education, and hypertension. Abbreviations: *CI*, confidence interval; AUC, the area under the curve; NRI, net reclassification improvement; IDI, integrated discrimination improvement.

## Data Availability

The data presented in this study are available upon request from the corresponding author due to privacy and ethical reasons.

## Supplementary Materials

The following supporting information can be downloaded at https://www.mdpi.com/article/10.3390/bios16040214/s1, Supplemental methods; Table S1: The eigenvalue and FVE for FPCs of noninvasive and capillary glucose.; Table S2: Characteristics comparison between training and validation sets for T2DM modeling; Table S3: Results of screening models for T2DM risk; Table S4: Performance of noninvasive and capillary screening models based on repeated cross-validation; Table S5: Comparison of noninvasive and capillary screening models (*n* = 125); Table S6: Characteristics comparison between training and validation sets for prediabetes modeling; Table S7: Results of the screening model for prediabetes risk; Figure S1: Multi-time-point blood glucose data density; Figure S2: Clarke error grid analysis for paired fasting glucose measurements; Figure S3: Subgroup analyses of equivalence testing; Figure S4: LASSO regression coefficient paths and cross-validation for optimal lambda selection for T2DM modeling; Figure S5: LASSO regression coefficient paths and cross-validation for optimal lambda selection for prediabetes modeling.

## Abbreviations

The following abbreviations are used in this manuscript:

CGM	Continuous glucose monitoring
DCA	Decision curve analysis
IDI	Integrated discrimination improvement
ISF	Interstitial fluid
MARD	Mean absolute relative difference
NIGM	Noninvasive glucose monitoring
NRI	Net reclassification improvement
RI	Reverse-iontophoresis
RIOSE	Reverse iontophoresis with on-skin electrochemistry
SHAP	SHapley Additive exPlanations
T2DM	Type 2 diabetes mellitus
