# Supplementary material for: A Flexible Wearable Glucose Sensor for Noninvasive Diabetes Screening: Functional Equivalence and Model Interpretability
Source: Biosensors (Basel). 2026 Apr 10;16(4):214. doi: 10.3390/bios16040214 (PMC13113994; doi:10.3390/bios16040214)
Supplement: Supplementary file 1 [file biosensors-16-00214-s001.zip › biosensors-4167773-supplementary.pdf]

A Flexible Wearable Glucose Sensor for Noninvasive Diabetes Screening: Functional Equivalence and Model Interpretability

Supplemental methods

Data collection, assessment, and quality control procedures

Covariates included sociodemographic factors, lifestyle behaviors, anthropometric measures, disease history, and laboratory factors. Sociodemographic factors comprised age (years), sex (male or female), marital status (married or others), education (secondary or below, undergraduate or vocational, and postgraduate), and ethnicity (Han or other ethnic minorities). Lifestyle behaviors included smoking status (yes or no), alcohol consumption (yes or no), sleep duration (7-8 hours/day or others), napping frequency (≤2 days/week or ≥3 days/week), habitual staying up late (never, sometimes, or always), regular exercise (never, sometimes, or always), and sweet preference (yes or no). Smoking status classified current and former smokers as "smokers", and lifelong non-smokers as "non-smokers". Alcohol consumption was defined as drinking ≥3 times per week. Sweet preference reflected habitual consumption of sweet-tasting foods or sugar-sweetened beverages. Anthropometric measurements included body mass index (BMI, kg/m^2), which was calculated as weight (kg) divided by height squared (m^2), waist-to-hip ratio (WHR), systolic blood pressure (mmHg), diastolic blood pressure (mmHg), and resting pulse (beats per minute). Disease history variables included self-reported hypertension and family history of T2DM (yes or no). Hypertension status (yes or no) was determined by the combination of self-reported history and objective blood pressure measurements. Laboratory measurements included glucose and HbA_{1c}.

Participants were also asked to report adverse events or any device-related discomfort during the trial period. Adverse events are defined as any unfavorable or unintended sign, symptom, or medical occurrence temporally associated with the use of the device or study procedures, regardless of causality. User comfort was assessed using a

structured five-point scale—“very comfortable”, “comfortable”, “no noticeable sensation”, “uncomfortable”, and “allergy/pain”—with an optional free-text field for additional comments. For analysis, “very comfortable”, “comfortable”, and “no noticeable sensation” were combined as “overall comfort”, and others were classified as “discomfort”.

To ensure data reliability, all blood glucose measurements were performed by trained medical personnel in accordance with standardized operating protocols. All glucose-measuring devices were calibrated before use according to the manufacturer's instructions. Data were examined for completeness and consistency before analysis. Two investigators independently reviewed implausible glucose values and extreme outliers, and participants with technically invalid measurements were excluded before final analyses.

Analytical accuracy of fasting glucose

Analytical accuracy was quantified by the mean absolute relative difference (MARD) and by the proportion of noninvasive glucose values falling within prespecified tolerance limits of the venous reference. MARD was calculated using the following equation: $\mathrm{MARD}=\frac{1}{n}\sum_{i=1}^{n}\left|\frac{\mathrm{Glu}_{test}-\mathrm{Glu}_{ref}}{\mathrm{Glu}_{ref}}\right|\times 100\%$, where n represents the total number of paired observations (n = 135), Glu_{test} is the noninvasive fasting glucose value, and Glu_{ref} is the corresponding venous reference glucose value.

Functional equivalence testing

We employed the functional equivalence test proposed by Holger and Kevin (2021)[1] to assess whether the noninvasive and capillary glucose curves are statistically equivalent over the monitoring period. Let $X_{1i}(t)$ and $X_{2i}(t)$ denote the noninvasive and capillary glucose functions of subject i over the period t. The mean functions are $\mu_1(t)$ and $\mu_2(t)$, with a difference of $\theta(t)=\mu_1(t)-\mu_2(t)$.

Pointwise equivalence limits were defined according to the capillary glucose:

$$\kappa_L(t)=\begin{cases}-1.1\text{mmol/L}, & \mu_2(t)<5.6\text{mmol/L}\\ -20\%\mu_2(t), & \mu_2(t)\geq 5.6\text{mmol/L}\end{cases} \quad \kappa_U(t)=\begin{cases}1.1\text{mmol/L}, & \mu_2(t)<5.6\text{mmol/L}\\ 20\%\mu_2(t), & \mu_2(t)\geq 5.6\text{mmol/L}\end{cases}$$

Following the maximal deviation framework, the test statistic was defined as $T_\theta = \max\left\{\sup_t\left[-\theta(t)+\kappa_L(t)\right],\sup_t\left[\theta(t)-\kappa_U(t)\right]\right\}$. Equivalence corresponds to $T_\theta < 0$, meaning the entire difference curve lies within the equivalence bounds.

To identify regions contributing to the maximum deviation, the extremal sets were estimated by

$$E_\theta^L=\left\{t:-\hat{\theta}_n(t)-\kappa_L(t)\geq \hat{T}_n^\theta - c\frac{\log(n)}{\sqrt{n}}\right\} \quad E_\theta^U=\left\{t:\hat{\theta}_n(t)-\kappa_U(t)\geq \hat{T}_n^\theta - c\frac{\log(n)}{\sqrt{n}}\right\}$$

with c = 0.005 and n = 135 subjects.

Paired bootstrap resampling was conducted at the subject level to preserve within-subject dependence. Let $D_i(t)=X_{1i}(t)-X_{2i}(t)$ and $\widetilde{D}_i(t)=D_i(t)-\overline{D}_i(t)$. For bootstrap iteration r: $Z_r^*(t)=\sqrt{n}\,\overline{D}_r^*(t)$, $T_\theta^{*(r)}=\max\left\{\sup_{t\in E_L}\left[-Z_r^*(t)\right],\sup_{t\in E_U}\left[Z_r^*(t)\right]\right\}$.

Let c_α be the empirical 5th percentile of $\left\{T_\theta^{*(r)}\right\}_{r=1}^R$ with R = 1,000. Functional equivalence was declared when $\sqrt{n}T_\theta < c_\alpha$.

Supplemental Reference

1. Dette H, Kokot K. Bio-equivalence tests in functional data by maximum deviation. *Biometrika*. 2020;108(4):895-913. DOI:10.1093/biomet/asaa096.

Table S1. The eigenvalue and FVE for FPCs of noninvasive and capillary glucose.

FPC	Noninvasive glucose		Capillary glucose	
	Eigenvalue	FVE (%)	Eigenvalue	FVE (%)
FPC 1	883.97	73.59	554.23	93.21
FPC 2	173.53	88.03	28.22	97.95
FPC 3	100.75	96.42	7.83	99.27
FPC 4	28.23	98.78	-	-
FPC 5	13.11	99.86	-	-

Abbreviations: FVE, fraction of variance explained; FPC, functional principal component.

Table S2. Characteristic comparison between training and validation sets for T2DM modeling.

Characteristics	Training (*n*=95)	Validation (*n*=40)	*P* value
Age, years	36.47 (15.00)	32.65 (14.06)	0.171
Sex			0.178
Male	34 (35.79)	20 (50.00)	
Female	61 (64.21)	20 (50.00)	
Marital status			0.336
Married	41 (43.16)	13 (32.50)	
Other	54 (56.84)	27 (67.50)	
Education			0.508
Secondary or below	14 (14.74)	6 (15.00)	
Undergraduate or vocational	50 (52.63)	17 (42.50)	
Postgraduate	31 (32.63)	17 (42.50)	
Ethnicity			>0.999
Han	87 (91.58)	37 (92.50)	
Other ethnic minorities	8 (8.42)	3 (7.50)	
Smoking			>0.999
Yes	8 (8.42)	4 (10.00)	
No	87 (91.58)	36 (90.00)	
Alcohol consumption			0.799
Yes	5 (5.26)	1 (2.50)	
No	90 (94.74)	39 (97.50)	
Sleep duration			0.283
7-8 hours per day	53 (55.79)	27 (67.50)	
Others	42 (44.21)	13 (32.50)	
Napping			0.067
≤2 days per week	65 (68.42)	20 (50.00)	
≥3 days per week	30 (31.58)	20 (50.00)	
Staying up status			0.220
Never	27 (28.42)	7 (17.50)	
Sometimes	26 (27.37)	9 (22.50)	
Always	42 (44.21)	24 (60.00)	
Exercise			0.269
Never	21 (22.11)	6 (15.00)	
Sometimes	38 (40.00)	22 (55.00)	
Always	36 (37.89)	12 (30.00)	
Sweet preference			0.516
Yes	24 (25.26)	13 (32.50)	
No	71 (74.74)	27 (67.50)	
Venous glucose, mmol/L	5.08 (0.91)	5.16 (1.03)	0.672
Noninvasive glucose, mmol/L	5.14 (1.29)	5.05 (1.04)	0.684
HbA_{1c}, %	5.74 (0.71)	5.98 (1.22)	0.149

BMI, kg/m^2	23.22 (5.73)	23.43 (4.80)	0.839
WHR, cm/cm	0.83 (0.09)	0.81 (0.09)	0.326
Pulse, bpm	72.85 (9.17)	74.58 (9.86)	0.331
Hypertension			0.973
Yes	10 (10.53)	5 (12.50)	
No	85 (89.47)	35 (87.50)	
Family history of T2DM			>0.999
Yes	8 (8.42)	4 (10.00)	
No	87 (91.58)	36 (90.00)	
Perceived comfort of the device			0.454
Overall comfort	75 (78.95)	35 (87.50)	
Discomfort	7 (7.37)	1 (2.50)	
Missing	13 (13.68)	4 (10.00)	

Notes: Data are presented as mean ± SD or *n* (%). Abbreviations: T2DM, type 2 diabetes mellitus; BMI, body mass index; WHR, waist-to-hip ratio.

Table S3. Results of screening models for T2DM risk.

	Noninvasive glucose		Capillary glucose	
	**Training**	**Validation**	**Training**	**Validation**
**AUC**	0.919 (95%*CI*: 0.847 - 0.991)	0.906 (95%*CI*: 0.800 - 1.000)	0.946 (95%*CI*: 0.891 - 1.000)	0.850 (95%*CI*: 0.706 - 0.993)
**Accuracy**	0.874 (95%*CI*: 0.790 - 0.933)	0.900 (95%*CI*: 0.763 - 0.972)	0.800 (95%*CI*: 0.705 - 0.875)	0.850 (95%*CI*: 0.702 - 0.943)
**Sensitivity**	0.900 (95%*CI*: 0.555 - 0.997)	0.909 (95%*CI*: 0.587 - 0.998)	1.000 (95%*CI*: 0.692 - 1.000)	0.909 (95%*CI*: 0.587 - 0.998)
**Specificity**	0.871 (95%*CI*: 0.780 - 0.934)	0.897 (95%*CI*: 0.726 - 0.978)	0.776 (95%*CI*: 0.673 - 0.860)	0.828 (95%*CI*: 0.642 - 0.942)
**PPV**	0.450 (95%*CI*: 0.231 - 0.685)	0.769 (95%*CI*: 0.462 - 0.738)	0.345 (95%*CI*: 0.179 - 0.543)	0.667 (95%*CI*: 0.702 - 0.882)
**NPV**	0.987 (95%*CI*: 0.928 - 1.000)	0.963 (95%*CI*: 0.810 - 0.999)	1.000 (95%*CI*: 0.946 - 1.000)	0.960 (95%*CI*: 0.384 - 0.999)
**Kappa**	0.535 (95%*CI*: 0.313 - 0.757)	0.763 (95%*CI*: 0.544 - 0.981)	0.422 (95%*CI*: 0.233 - 0.612)	0.662 (95%*CI*: 0.662 - 0.904)

Notes: the models included noninvasive fasting glucose or capillary fasting glucose, alongside age, education, and hypertension. Abbreviations: T2DM, type 2 diabetes mellitus; AUC, the area under the curve; *CI*, confidence interval; PPV, positive predictive value; and NPV, negative predictive value.

Table S4. Performance of noninvasive and capillary screening models based on repeated cross-validation.

	Noninvasive model	Capillary model
**AUC**	0.844 (95%*CI*: 0.814 - 0.873)	0.830 (95%*CI*: 0.797 - 0.863)
**Accuracy**	0.727 (95%*CI*: 0.702 - 0.750)	0.729 (95%*CI*: 0.704 - 0.752)
**Sensitivity**	0.924 (95%*CI*: 0.879 - 0.956)	0.790 (95%*CI*: 0.729 - 0.843)
**Specificity**	0.690 (95%*CI*: 0.663 - 0.717)	0.718 (95%*CI*: 0.690 - 0.744)
**PPV**	0.355 (95%*CI*: 0.315 - 0.396)	0.340 (95%*CI*: 0.298 - 0.384)
**NPV**	0.980 (95%*CI*: 0.968 - 0.989)	0.949 (95%*CI*: 0.932 - 0.963)
**Kappa**	0.371 (95%*CI*: 0.327 - 0.416)	0.330 (95%*CI*: 0.281 - 0.379)

Notes: the models included noninvasive fasting glucose or capillary fasting glucose, alongside age, education, and hypertension. Abbreviations: T2DM, type 2 diabetes mellitus; AUC, the area under the curve; *CI*, confidence interval; PPV, positive predictive value; and NPV, negative predictive value.

Table S5. Comparison of noninvasive and capillary screening models (*n* = 125).

	Estimate	95% *CI*	*P* value
**AUC**			0.619
Capillary model	0.772	0.614 - 0.930	
Noninvasive model	0.794	0.677 - 0.912	
**NRI**			0.295
Capillary model	Ref.		
Noninvasive model	0.044	-0.038 - 0.126	
**IDI**			0.439
Capillary model	Ref.		
Noninvasive model	-0.027	-0.096 - 0.042	

Notes: the capillary and noninvasive models included fasting glucose, age, education, and hypertension. Abbreviations: *CI*, confidence interval; AUC, the area under the curve; NRI, net reclassification improvement; and IDI, integrated discrimination improvement.

Table S6. Characteristic comparison between training and validation sets for prediabetes modeling.

Characteristics	Training (*n* = 80)	Validation (*n* = 34)	*P* value
Age, years	32.67 (13.51)	32.56 (11.03)	0.965
Sex			0.270
Male	34 (42.50)	10 (29.41)	
Female	46 (57.50)	24 (70.59)	
Marital status			0.420
Married	25 (31.25)	14 (41.18)	
Other	55 (68.75)	20 (58.82)	
Education			0.503
Secondary or below	5 (6.25)	4 (11.76)	
Undergraduate or vocational	40 (50.00)	18 (52.94)	
Postgraduate	35 (43.75)	12 (35.29)	
Ethnicity			0.372
Han	76 (95.00)	30 (88.24)	
Other ethnic minorities	4 (5.00)	4 (11.76)	
Smoking			>0.999
Yest	7 (8.75)	3 (8.82)	
No	73 (91.25)	31 (91.18)	
Alcohol consumption			0.993
Yes	3 (3.75)	2 (5.88)	
No	77 (96.25)	32 (94.12)	
Sleep duration			0.302
7-8 hours per day	50 (62.50)	17 (50.00)	
Others	30 (37.50)	17 (50.00)	
Napping			0.128
≤2 days per week	45 (56.25)	25 (73.53)	
≥3 days per week	35 (43.75)	9 (26.47)	
Staying up status			0.541
Never	18 (22.50)	5 (14.71)	
Sometimes	23 (28.75)	9 (26.47)	
Always	39 (48.75)	20 (58.82)	
Exercise			0.132
Never	14 (17.50)	10 (29.41)	
Sometimes	39 (48.75)	10 (29.41)	
Always	27 (33.75)	14 (41.18)	
Sweet preference			0.463
Yes	26 (32.50)	8 (23.53)	
No	54 (67.50)	26 (76.47)	
Venous glucose, mmol/L	4.87 (0.49)	4.81 (0.35)	0.463
Noninvasive glucose, mmol/L	5.03 (1.29)	4.79 (0.89)	0.324

HbA_{1c}, %	5.54 (0.44)	5.49 (0.41)	0.594
BMI, kg/m^2	23.46 (5.90)	21.63 (3.69)	0.098
WHR, cm/cm	0.83 (0.10)	0.81 (0.07)	0.274
Pulse, bpm	73.62 (9.72)	73.71 (9.73)	0.968
Hypertension			0.372
Yes	4 (5.00)	4 (11.76)	
No	76 (95.00)	30 (88.24)	
Family history of T2DM			0.372
Yes	4 (5.00)	4 (11.76)	
No	76 (95.00)	30 (88.24)	
Perceived comfort of the device			0.802
Overall comfort	65 (81.25)	29 (85.29)	
Discomfort	5 (6.25)	2 (5.88)	
Missing	10 (12.5)	3 (8.83)	

Notes: Data are presented as mean ± SD or *n* (%). Abbreviations: T2DM, type 2 diabetes mellitus; BMI, body mass index; and WHR, waist-to-hip ratio.

Table S7. Results of the screening model for prediabetes risk.

	Training	Validation
**AUC**	0.784 (95%*CI*: 0.683 - 0.885)	0.760 (95%*CI*: 0.572 - 0.948)
**Accuracy**	0.713 (95%*CI*: 0.600 - 0.808)	0.735 (95%*CI*: 0.556 - 0.871)
**Sensitivity**	0.824 (95%*CI*: 0.655 - 0.932)	0.889 (95%*CI*: 0.518 - 0.997)
**Specificity**	0.630 (95%*CI*: 0.475 - 0.768)	0.680 (95%*CI*: 0.465 - 0.851)
**PPV**	0.622 (95%*CI*: 0.465 - 0.762)	0.500 (95%*CI*: 0.247 - 0.753)
**NPV**	0.829 (95%*CI*: 0.664 - 0.934)	0.944 (95%*CI*: 0.727 - 0.999)
**Kappa**	0.436 (95%*CI*: 0.247 - 0.624)	0.456 (95%*CI*: 0.180 - 0.731)

Notes: the models included second and third principal components of noninvasive glucose, age, hypertension, ethnicity, and alcohol consumption. Abbreviations: AUC, the area under the curve; *CI*, confidence interval; PPV, positive predictive value; and NPV, negative predictive value.

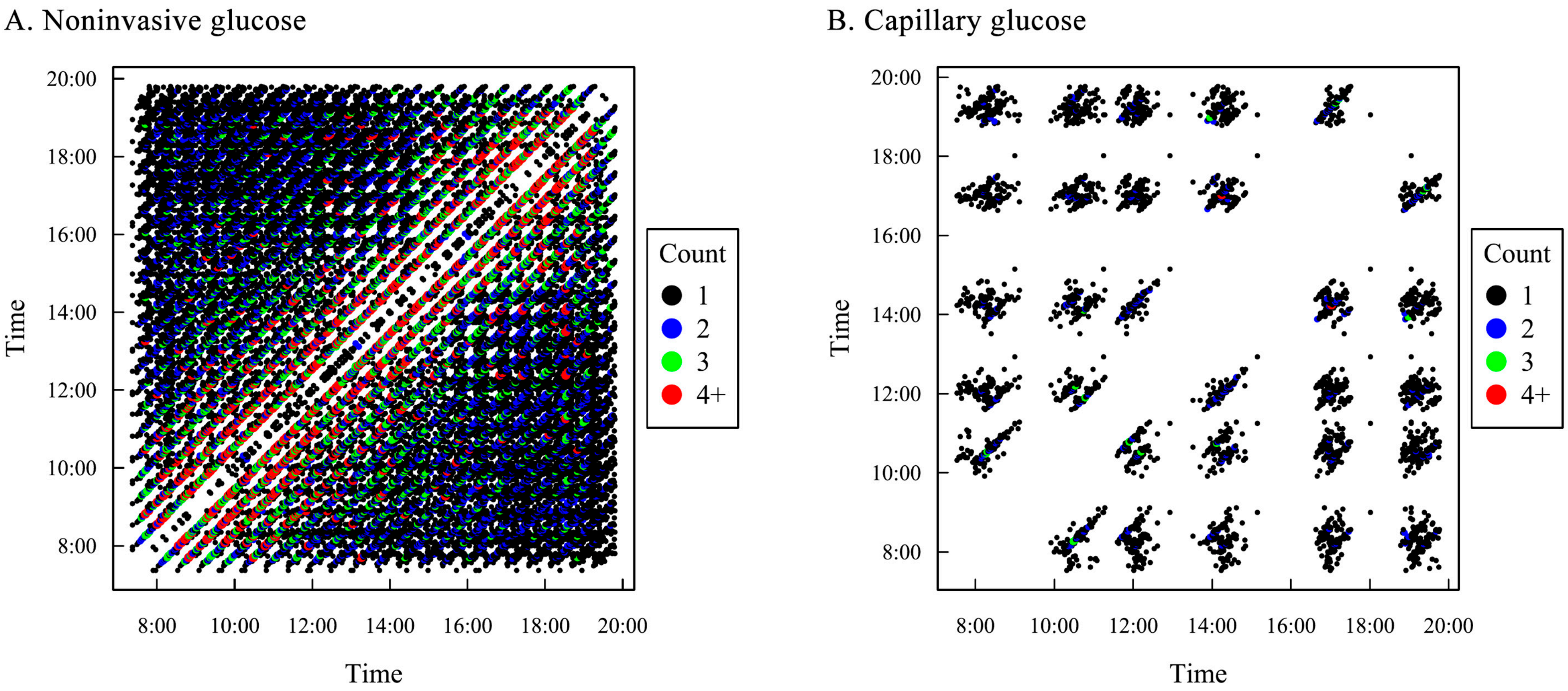

Figure S1. Multi-timepoint blood glucose data density.

Notes: A was the time distribution of noninvasive glucose, and B was the time distribution of capillary glucose.

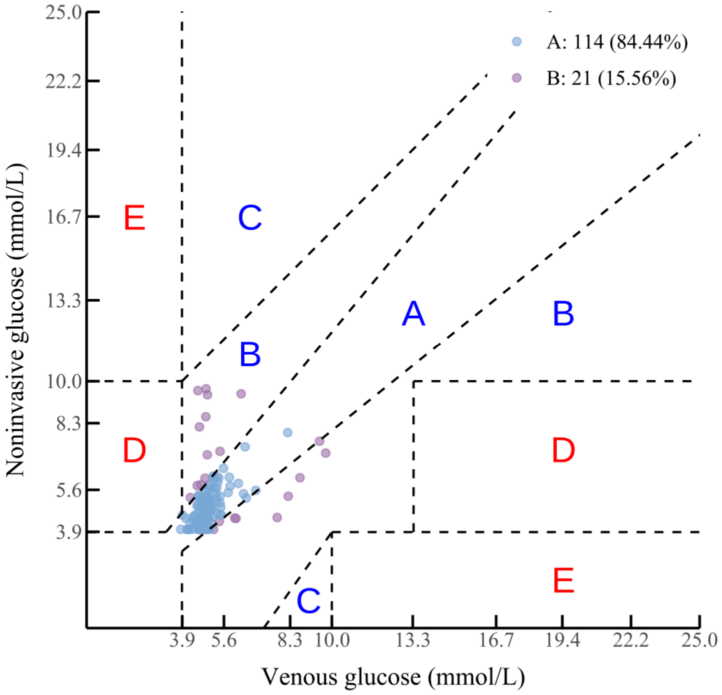

Figure S2. Clarke error grid analysis for paired fasting glucose measurements.

Notes: The error grid displays 135 paired observations. The reference venous glucose values are plotted on the x-axis, and the noninvasive glucose values are plotted on the y-axis. Zones A and B are clinically acceptable.

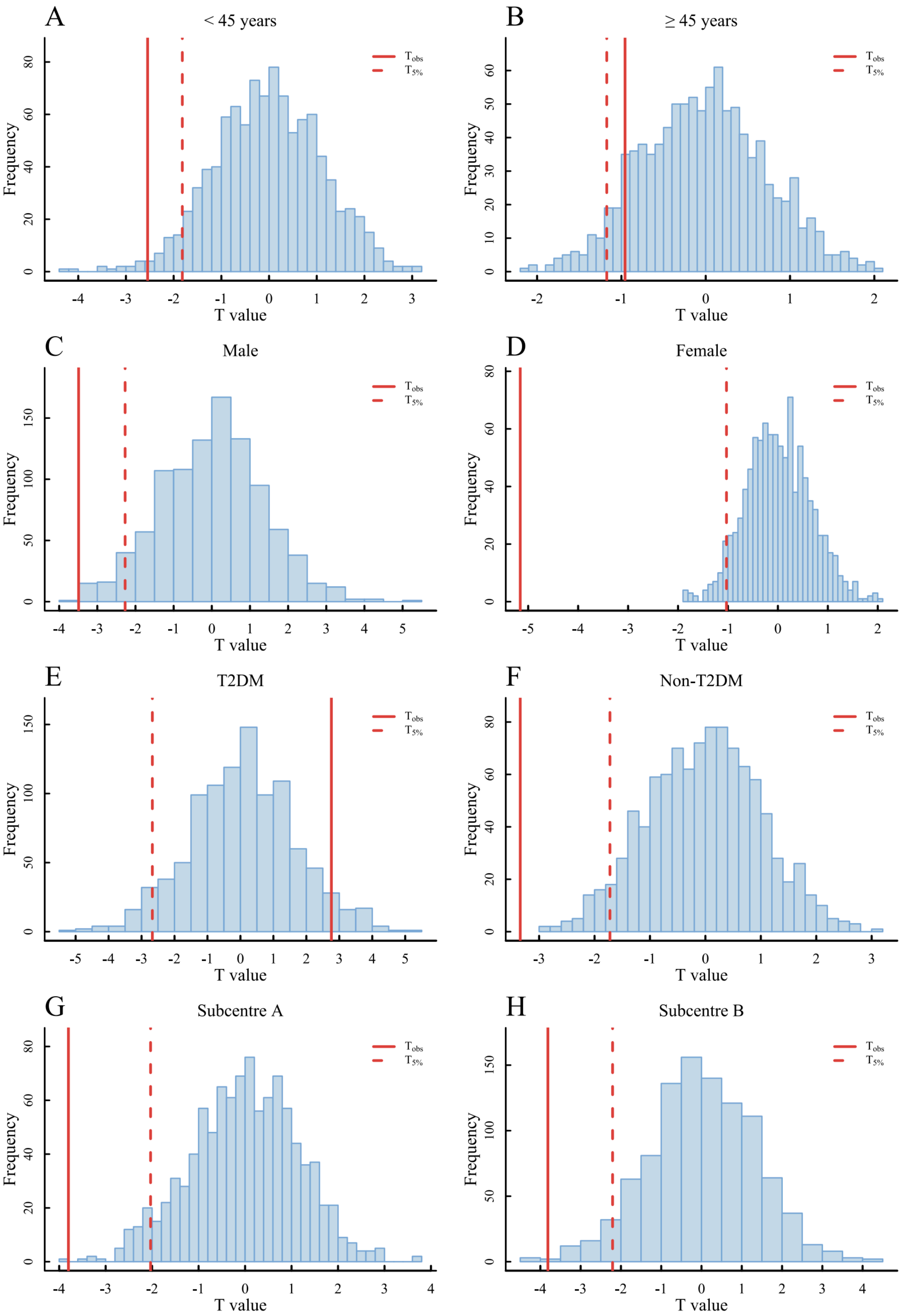

Figure S3. Subgroup analyses of equivalence testing.

Notes: Equivalence testing was conducted within the subgroups of age (<45 *vs*. ≥45 years), sex (male *vs*. female), T2DM status (yes *vs*. no), and trial site (subcenter A *vs*. subcenter B). Abbreviations: T2DM, type 2 diabetes mellitus.

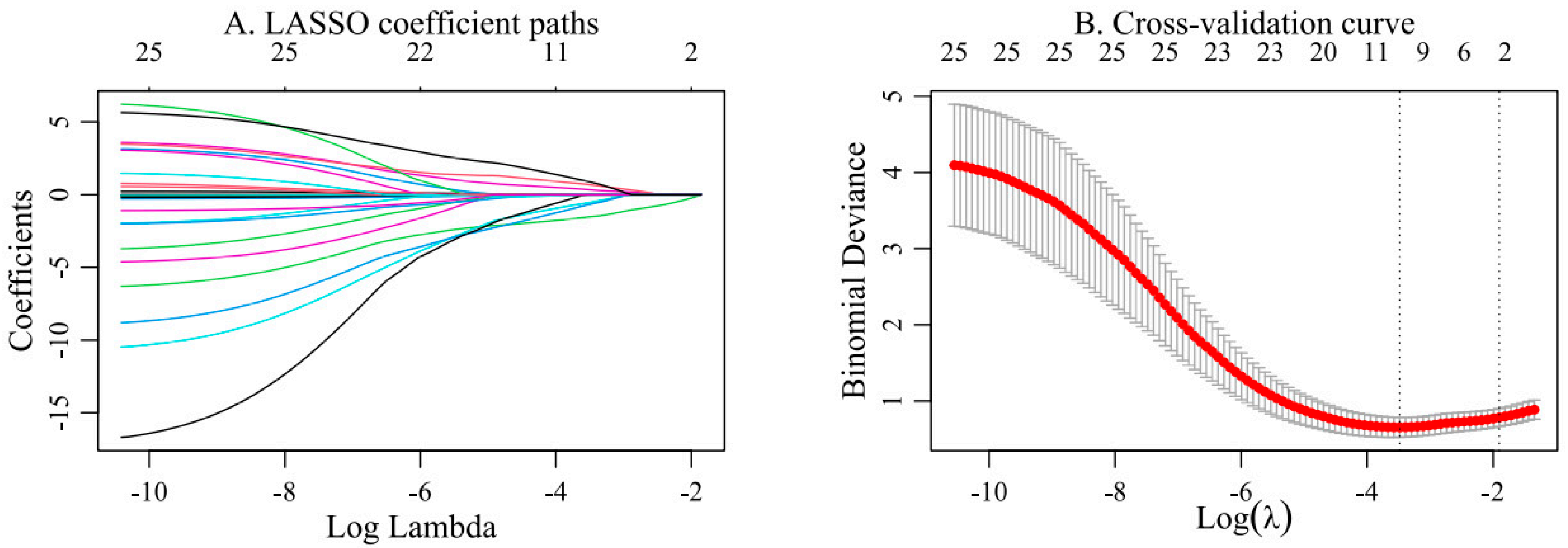

Figure S4. LASSO regression coefficient paths and cross-validation for optimal lambda selection for T2DM modeling.

Notes: A is the coefficient path of LASSO regression, and B is the cross-validation curve. Abbreviations: LASSO, least absolute shrinkage and selection operator.

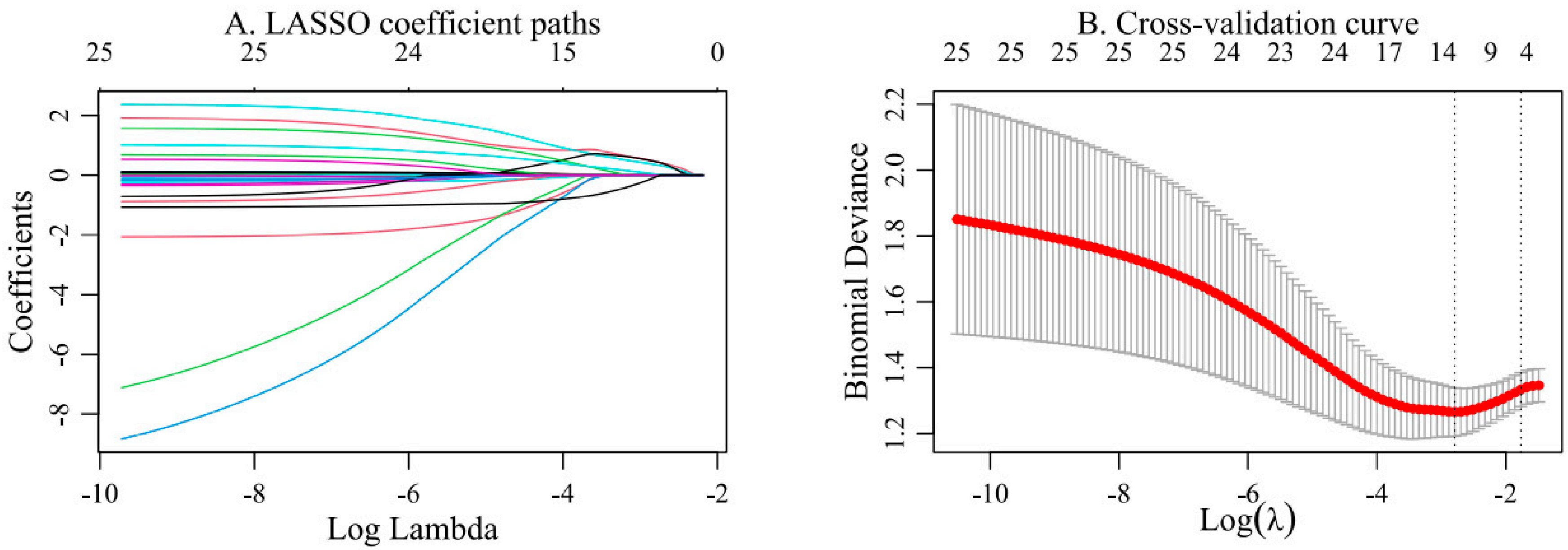

Figure S5. LASSO regression coefficient paths and cross-validation for optimal lambda selection for prediabetes modeling.

Notes: A is the coefficient path of LASSO regression, and B is the cross-validation curve. Abbreviations: LASSO, least absolute shrinkage and selection operator.
